# Caspase-11 Mediates Neutrophil Chemotaxis and Extracellular Trap Formation During Acute Gouty Arthritis Through Alteration of Cofilin Phosphorylation

**DOI:** 10.3389/fimmu.2019.02519

**Published:** 2019-11-15

**Authors:** Kyle Caution, Nicholas Young, Frank Robledo-Avila, Kathrin Krause, Arwa Abu Khweek, Kaitlin Hamilton, Asmaa Badr, Anup Vaidya, Kylene Daily, Hawin Gosu, Midhun N. K. Anne, Mostafa Eltobgy, Duaa Dakhlallah, Sudha Argwal, Shady Estfanous, Xiaoli Zhang, Santiago Partida-Sanchez, Mikhail A. Gavrilin, Wael N. Jarjour, Amal O. Amer

**Affiliations:** ^1^Department of Microbial Infection and Immunity, The Ohio State University Medical Center, Columbus, OH, United States; ^2^Department of Rheumatology and Immunology, The Ohio State University Medical Center, Columbus, OH, United States; ^3^Center for Microbial Pathogenesis, Nationwide Children's Hospital, Columbus, OH, United States; ^4^Department of Biology and Biochemistry, Birzeit University, West Bank, Palestine; ^5^Department of Microbiology, Immunology and Cell Biology, West Virginia University, Morgantown, WV, United States; ^6^Center for Biostatistics, The Ohio State University Medical Center, Columbus, OH, United States; ^7^Department of Internal Medicine, The Ohio State University Medical Center, Columbus, OH, United States

**Keywords:** caspase-11, gout, neutrophils, macrophages, NETosis, IL-1β, cell migration, inflammasome

## Abstract

Gout is characterized by attacks of arthritis with hyperuricemia and monosodium urate (MSU) crystal-induced inflammation within joints. Innate immune responses are the primary drivers for tissue destruction and inflammation in gout. MSU crystals engage the Nlrp3 inflammasome, leading to the activation of caspase-1 and production of IL-1β and IL-18 within gout-affected joints, promoting the influx of neutrophils and monocytes. Here, we show that *caspase-11*^−/−^ mice and their derived macrophages produce significantly reduced levels of gout-specific cytokines including IL-1β, TNFα, IL-6, and KC, while others like IFNγ and IL-12p70 are not altered. IL-1β induces the expression of caspase-11 in an IL-1 receptor-dependent manner in macrophages contributing to the priming of macrophages during sterile inflammation. The absence of caspase-11 reduced the ability of macrophages and neutrophils to migrate in response to exogenously injected KC *in vivo*. Notably, *in vitro, caspase-11*^−/−^ neutrophils displayed random migration in response to a KC gradient when compared to their WT counterparts. This phenotype was associated with altered cofilin phosphorylation. Unlike their wild-type counterparts, *caspase-11*^−/−^ neutrophils also failed to produce neutrophil extracellular traps (NETs) when treated with MSU. Together, this is the first report demonstrating that caspase-11 promotes neutrophil directional trafficking and function in an acute model of gout. Caspase-11 also governs the production of inflammasome-dependent and -independent cytokines from macrophages. Our results offer new, previously unrecognized functions for caspase-11 in macrophages and neutrophils that may apply to other neutrophil-mediated disease conditions besides gout.

## Introduction

Gout is an ancient disease that is estimated to affect 8.3 million people in the United States, and this number is steadily increasing (1). Gout, in its acute or chronic forms, occurs when the body mounts an inflammatory response against the crystalized form of uric acid, monosodium urate crystals (MSU), within joint spaces (2, 3).

The activation of inflammasomes requires two signals; one for priming and one for activation. This leads to inflammasome assembly, caspase-1 activation, maturation of IL-1β and IL-18, and sometimes pyroptosis via Gasdermin (GSDM) and the P2X7 receptor (4–7). Gout has been identified as a prototype IL-1β-dependent inflammatory disease (8, 9). IL-1β, an endogenous pyrogen, is first synthesized as an inactive precursor and then is cleaved to its bioactive form by caspase-1 that is canonically activated within the inflammasome complex (10–12). An intense inflammatory response is initiated when MSU crystals are phagocytosed by resident, primed macrophages and sensed by the NLR Family Pyrin Domain-Containing 3 (NLRP3), which is followed by the assembly of the inflammasome and caspase-1 activation (11). Inflammasome-dependent cytokines, interleukin-1 beta (IL-1β), and interleukin-18 (IL-18) are then released, activating interstitial cells and promoting influx of polymorphonuclear cells (PMNs—neutrophils), monocytes, and macrophages (11, 13). Release of these cytokines in gout induces the differentiation of osteoclasts from mononuclear precursors and stimulates bone resorption (14, 15). This inflammatory microenvironment also promotes more neutrophil extravasation into the joint space (16, 17). Within the affected joint space, immune cells release other pro-inflammatory cytokines like TNFα, IL-6, and IL-8 (CXCL1/KC); eicosanoid lipid mediators; and production of the enzymes collagenase and stromelysin-1 that degrade the extracellular matrix in articular structures. This causes a cascade of cytotoxic cellular contents and danger-associated molecular patterns (DAMPs) to leak into extracellular venues and recruit other immune cells, producing more inflammation and tissue destruction (11, 18). MSU crystals have been found to activate these infiltrating PMNs, not only by triggering cytokine secretion, but also by inducing neutrophil extracellular traps (NETs) (19, 20). Neutrophils form NETs in response to various proinflammatory, infectious stimuli, or crystals including MSU (21). NET formation results in the release of nuclear DNA coated with neutrophil granule enzymes, to generate NETs (21, 22). NETs orchestrate the initiation and progression of inflammation in gout (8, 20, 23).

The role of caspase-11 is versatile according to the insult. Our group and others have demonstrated several non-apoptotic functions for caspase-11. We found that caspase-11 restricts *Legionella pneumophila* infection by promoting the activation of the RhoA GTPase–cofilin pathway in order to regulate actin dynamics and phagolysosomal fusion within the cell (24, 25). Caspase-11 also promotes autophagosome formation to control *Burkholderia cenocepacia* infection via regulation of Rab7 and actin dynamics (26). Yan's group has demonstrated that caspase-11 modulates cofilin via the actin interacting protein 1 (Aip1) to promote migration of T cells (27). We recently found that caspase-11 is actually exploited by methicillin-resistant *Staphylococcus aureus* (MRSA) to survive in macrophages (28). When endotoxin contaminates the intracellular spaces of macrophages, caspase-11 senses the LPS and promotes downstream activation of caspase-1 and IL-1β (29–31). In other circumstances, caspase-11 mediates the release, but not the activation of IL-1β (30, 32, 33). Interestingly, caspase-11 is also essential for the production of KC in response to infection with *B. cenocepacia* (26). These data demonstrate that, in macrophages, caspase-11 exerts essential immune functions independently of cell death. However, the role of caspase-11 in neutrophils is still enigmatic.

The role of caspase-11 in gout has not yet been investigated. In this study, we found that *caspase-11*^−/−^ mice exhibit significantly less signs of joint inflammation, including swelling and tissue damage upon injection of MSU crystals in the tibio-tarsal joint space *in vivo*. The absence of caspase-11 is associated with significantly less neutrophil influx and lower levels of inflammatory cytokines in the synovial fluid of MSU-injected joints. The reduction in neutrophil migration was not solely due to low cytokine production within the MSU-injected joint, but also due to an innate alteration in the ability of *caspase-11*^−/−^ neutrophils to migrate toward a chemotactic signal. Particularly, *caspase-11*^−/−^ neutrophils displayed a random migration pattern in response to a KC gradient when compared to their WT counterparts. In addition, *caspase-11*^−/−^ neutrophils produced significantly less NETs in response to MSU (16, 34, 35) independently of the receptor-interacting protein kinase 3 (RIPK3) and mixed lineage kinase domain-like (MLKL) pathway. This phenotype is independent of gasdermin (GSDM). In addition, we found that the phosphorylation of cofilin is altered in *caspase-11*^−/−^ neutrophils in response to MSU. Together, this is the first report demonstrating that caspase-11 promotes neutrophil directional trafficking and NET formation in an acute model of gout. Our results offer caspase-11 and its human orthologs, caspase 4 and 5, as viable targets in gout and other disease conditions that are driven by activated neutrophils.

## Results

### Swelling in the Tibio-tarsal Joints of Mice Upon MSU Injection Requires the Expression of Caspase-11

**Figure 1:**
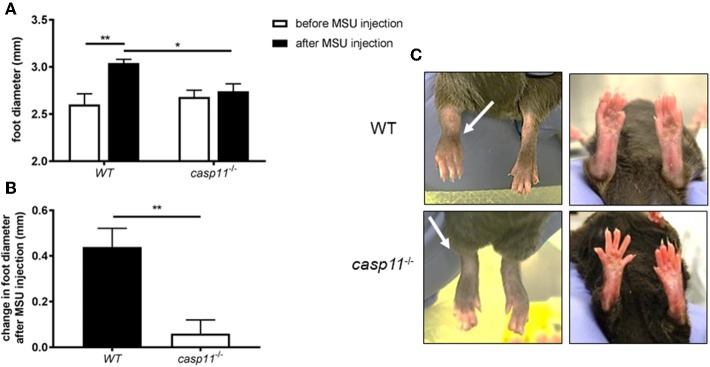
Swelling and increase in ankle diameter during acute gout requires caspase-11 expression. **(A)** Caliper measurement of WT and *caspase-11*^−/−^ ankle diameters before and after injection of 0.5 mg monosodium urate (MSU) crystals into the tibio-tarsal joint. A two-way ANOVA with a Holm's Sidak comparison test was performed for statistical analysis. **(B)** Caliper measurement before MSU injection subtracted from measurement after injection to depict change in ankle diameter. Student's *t* test analysis was performed. *p <0.05, **p <0.01, *n* = 5 mice. **(C)** Representative superior and inferior pictures of WT and *caspase-11*^−/−^ ankles 24 h post injection.

Gout is due to an acute or chronic inflammatory response to MSU deposition in the joint space (13, 36). This causes the classical signs of inflammation, including swelling, tenderness, heat, and redness. Meanwhile, it has been shown that caspase-11 plays a role in promoting inflammation and cell death in response to PAMPs (pathogen-associated molecular patterns) and DAMPs (37–39). To determine if caspase-11 contributes to the classical signs of inflammation during gout, WT and *caspase-11*^−/−^ mice were injected with MSU crystals into the tibio-tarsal joint of the right leg. After 24 h, the affected ankle joints were examined. We found that MSU injection promotes acute swelling in joints of WT but not *caspase-11*^−/−^ mice. Caliper measurements demonstrated that WT mice exhibited significantly increased joint diameter size when compared to *caspase-11*^−/−^ mice after MSU injection (Figure 1A). In addition, the joint diameter after MSU injection was significantly increased in WT mice, while the joint diameter in the *caspase-11*^−/−^ mice remained unchanged after MSU injection (Figure 1B). Accordingly, MSU injection caused more punctuated redness in the footpad, ankle, and leg of the WT mice when compared to *caspase-11*^−/−^ counterparts (Figure 1C). Vehicle (PBS) injection in mice did not induce swelling, as quantified by caliper, or visible signs of inflammation, demonstrating that this response was specific to MSU (Supplementary Figures 1A–C). Real-time quantitative (RT) PCR analysis performed on RNA isolated from joint samples revealed that MSU significantly induced the expression of caspase-11 mRNA *in vivo*, compared to the vehicle (PBS)-treated joints (Supplementary Figure 2). Taken together, these data indicate that caspase-11 is required for the swelling in the tibio-tarsal joints using an acute gout mouse model.

### The Absence of Caspase-11 Is Associated With Reduced Immune Cell Influx and Less Joint Damage *in vivo* in Response to MSU

**Figure 2:**
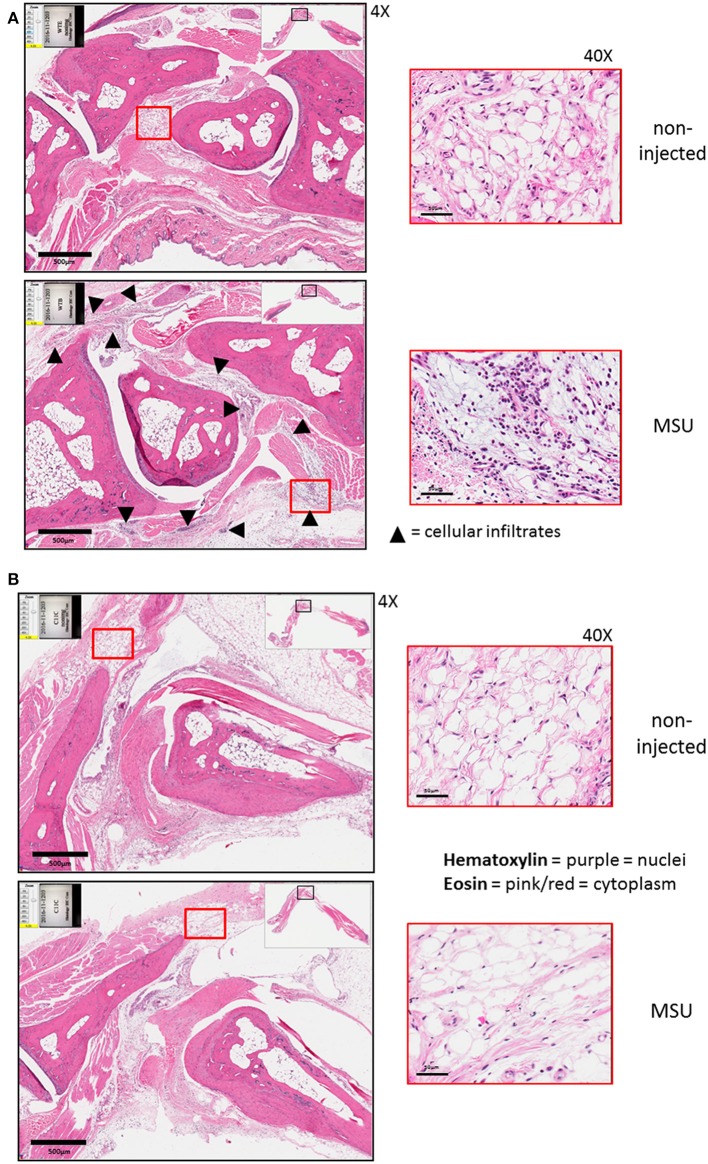
Histological tissue changes in response to MSU injection require the expression of caspase-11. **(A)** Aperio images of hematoxylin and eosin (H&E) staining of WT ankle joints 24 h post injection with PBS or MSU. Arrowheads on 4 × images indicate cellular infiltrates. 40 × magnifications depict markedly more hematoxylin staining. **(B)** Aperio images of H&E staining of *caspase-11*^−/−^ ankle joints 24 h post injection with PBS or MSU. 4 × and 40 × magnifications show lack of cellular infiltrates (arrowheads), as PBS and MSU images look histologically similar. Images are representative from each group of five mice.

**Figure 3:**
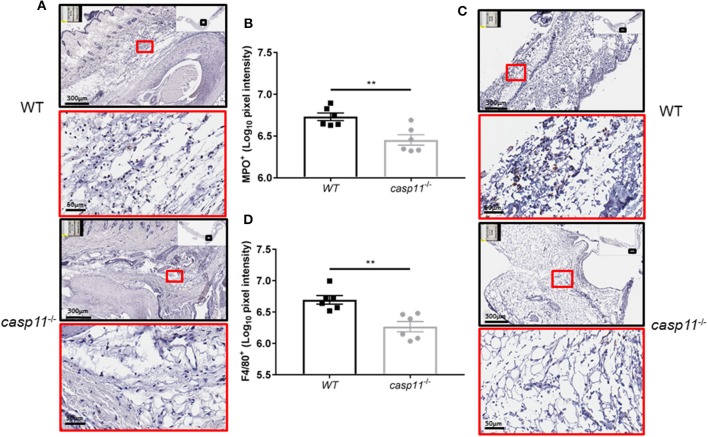
Caspase-11 promotes neutrophil and macrophage influx in response to MSU *in vivo*. **(A)** Representative Aperio images of myeloperoxidase (MPO) DAB staining of WT and *caspase-11*^−/−^ ankle joints 24 h post injection with MSU. Black boxes are 8 × magnification and red boxes are 40 × magnification. Brown staining indicates infiltrating neutrophils (PMNs) within the joint space. **(B)** Quantification of WT and *caspase-11*^−/−^ MPO DAB staining of 40 × magnification via Aperio analysis software. **p <0.01, *n* = 5 mice. **(C)** Representative Aperio images of F4/80 DAB staining of WT and *caspase-11*^−/−^ ankle joints 24 h post injection with MSU. Black boxes are 8 × magnification and red boxes are 40 × magnification. Brown staining indicates infiltrating macrophages within the joint space. **(D)** Quantification of WT and *caspase-11*^−/−^ MPO DAB staining of 40 × magnification via Aperio analysis software. **p <0.01, *n* = 5 mice. **(B,D)** Student's *t* test analysis was performed.

The expression of caspase-11 mediates swelling of MSU-treated joints (Figure 1). To determine if caspase-11 plays a role in the cellular influx and/or overall structural changes in our acute gout model, we injected MSU crystals into the right tibio-tarsal joint of WT and *caspase-11*^−/−^ mice. High-resolution images of hematoxylin and eosin (H&E) slides were generated using the Aperio ImageScope™ (Leica Biosystems) technology and assessed by a pathologist in the Ohio State University Comparative Pathology and Mouse Phenotyping core. H&E analysis of the synovial area demonstrated that joints of *caspase-11*^−/−^ mice contained less immune cells within the deep dermis, peri-articular soft tissue, and areas subjacent to the periosteal bone of the joint (Figures 2A,B). Signs of necrosis and damage were also blunted in the *caspase-11*^−/−^ mice as tissue organization remained intact, while their WT counterparts displayed greater signs of tissue destruction (Figures 2A,B). Because H&E analysis indicated that *caspase-11*^−/−^ mice exhibited impairment in cellular recruitment after MSU injection, we then determined the identity of the inflammatory cells extravasating into the MSU-injected joint by immunohistochemical assays on processed ankle joints 24 h after MSU injection. Slides prepared from WT and *caspase-11*^−/−^ mice were stained for myeloperoxidase (MPO) and F4/80 (Emr1) to assay neutrophil and macrophage influx, respectively, into the joint space. Immunohistological staining demonstrated that *caspase-11*^−/−^ mice exhibited a marked impairment in MPO^+^ and F4/80^+^ cell infiltration into the tibio-tarsal space when compared to their WT counterparts (Figures 3A,C). In addition, using high-resolution images, histological staining was quantified via the Aperio ImageScope software and showed that *caspase-11*^−/−^ mice displayed significantly less neutrophils and macrophages in comparison to the WT ankle joints (Figures 3B,D). In sum, these data demonstrate that caspase-11 is required for immune cell influx into the synovium that leads to tissue destruction and remodeling in the gout mouse model. This phenotype could be due to reduced production of cytokines in the absence of caspase-11, an inherent trafficking defect of immune cells or both.

### Caspase-11^−/−^ Mice Produce Significantly Less Gout-Specific Cytokines in Response to MSU Injection in Their Joints

**Figure 4:**
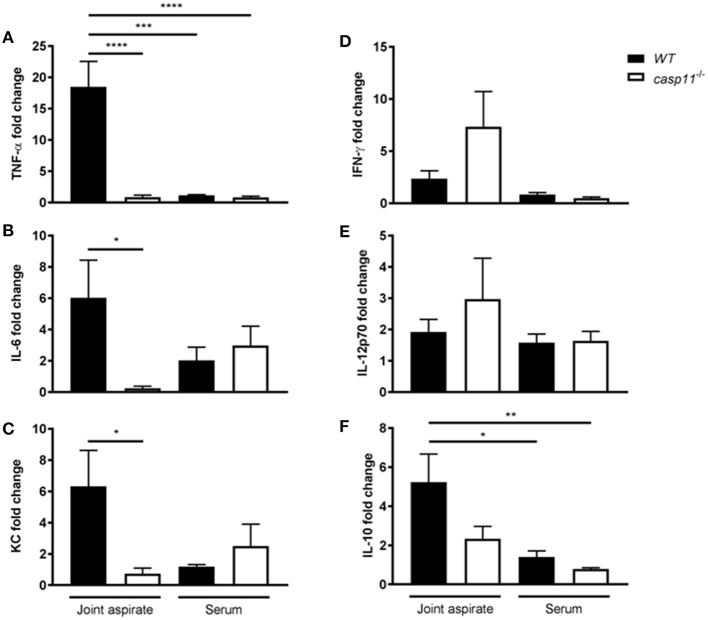
Gout-specific cytokines TNFα, IL-6, and KC are significantly upregulated in joints of WT but not *caspase-11*^−/−^ mice during acute gout. *In vivo* MULTI-ARRAY electrochemiluminescence ELISA of WT and *caspase-11*^−/−^ joint aspirate and serum cytokine fold change levels compared to PBS-treated controls. **(A)** TNFα, **(B)** IL-6, **(C)** KC, **(D)** IFNγ, **(E)** IL-12p70, and **(F)** IL-10. One-way ANOVA with Tukey's multi-comparison performed. *p <0.05, **p <0.01, ***p <0.001, ****p <0.0001, *n* = 5 mice.

MSU elicits a strong immune response via a host of pro-inflammatory cytokines. IL-1β plays a central role in the pathogenesis of gout, in addition to the other pro-inflammatory cytokines that are produced within the tissue microenvironment during a gout attack (40, 41). These cytokines, such as TNFα, IL-6, and CXCL1 (KC), are important in activating resident cells, inducing endothelial permeability and cellular infiltration, and promoting overall tissue remodeling (42). To determine if caspase-11 contributes to the production of inflammatory cytokines, WT and *caspase-11*^−/−^ mice were injected with MSU into the tibio-tarsal joint and synovial fluid and serum were collected after 24 h. Using Meso Scale Discovery's (MSD) electro-chemiluminescence (ECL) V-PLEX platform, we found that TNFα, IL-6, and KC were significantly reduced within the synovium of *caspase-11*^−/−^ mice when compared to that of WT (Figures 4A–C). Analysis from the sera of the mice, however, indicated that TNFα, IL-6, and KC levels were comparable between the two strains (Figures 4A–C). Therefore, the inflammatory response observed in the WT mice demonstrated that caspase-11 is needed for their secretion within MSU-injected joints. Conversely, non-gout cytokines, IFN-γ and IL-12p70 were similarly secreted into the synovial fluid of WT and *caspase-11*^−/−^ mice and equally low in the sera in both strains (Figures 4D,E). Since anti-inflammatory mechanisms are usually engaged whenever an inflammatory response occurs, we also tested levels of the classic anti-inflammatory mediator, IL-10. We found that IL-10 was reduced within the synovial fluid of *caspase-11*^−/−^ mice compared to that of WT mice and was comparable between the sera of the two strains (Figure 4F). Altogether, these data designate caspase-11 as a contributor to the release of TNFα, IL-6, and KC but not IFN-γ and IL-12p70 in the synovial fluid.

**Figure 5:**
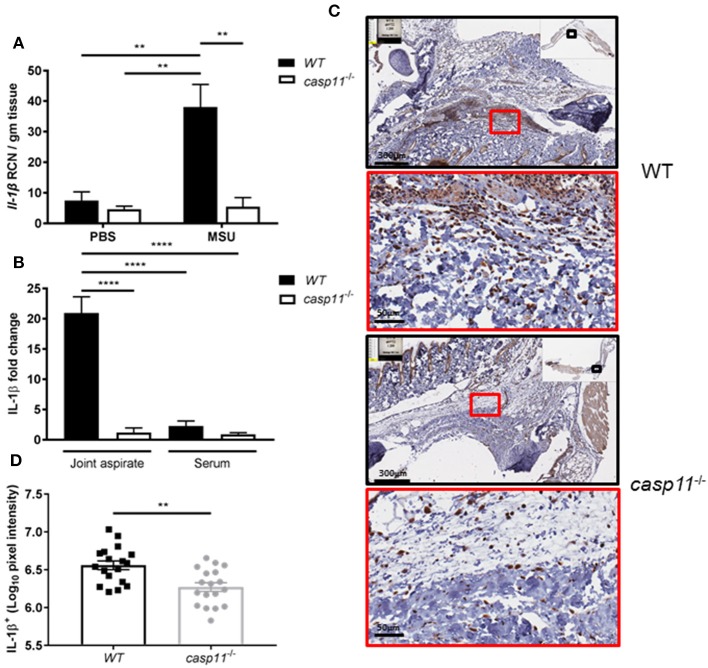
IL-1β is induced and secreted in a caspase-11-dependent manner during acute gout *in vivo*. **(A)** Relative copy number (RCN) per gram of tissue of *Il-1*β from WT and *caspase-11*^−/−^ joint tissue treated with vehicle or MSU. Two-way ANOVA with a Holm's Sidak *post-hoc* test was used for statistical analysis. **p <0.01, *n* = 5 mice. **(B)** *In vivo* MULTI-ARRAY electrochemiluminescence ELISA of joint aspirate and serum fold change levels of IL-1β from WT and *caspase-11*^−/−^ mice in response to MSU and compared to PBS-treated controls. Statistical analysis performed using a one-way ANOVA with Tukey's multiple comparisons. ****p <0.0001, *n* = 5 mice. **(C)** Representative Aperio images of IL-1β DAB staining of WT and *caspase-11*^−/−^ ankle joints 24 h post injection with MSU. Black boxes are 8 × magnification and red boxes are 40 × magnification. Brown staining indicates IL-1β-positive cells within the joint space. **(D)** Quantification of WT and *caspase-11*^−/−^ IL-1β DAB staining of 40 × magnification via Aperio analysis software. Student's *t* test was performed. **p <0.01, *n* = 5 mice.

Because IL-1β is a pivotal cytokine in the pathogenesis of gout and its presence designates the activation of the inflammasome, we determined the role of caspase-11 in the production of IL-1β within the synovium. We injected MSU into the tibio-tarsal joints of WT and *caspase-11*^−/−^ mice and isolated joint tissue after 24 h. Real-time quantitative PCR analysis performed on RNA isolated from joint samples revealed that *Il1b* expression was upregulated in WT mouse joints, whereas *caspase-11*^−/−^ mice expressed significantly less (Figure 5A). Furthermore, synovial fluid and serum were collected from the mice and analyzed for IL-1β secretion into the joint space and the circulation, respectively. In response to MSU injection, IL-1β secretion was significantly increased in the synovial fluid of the WT mice, but severely hindered in the *caspase-11*^−/−^ mice (Figure 5B). IL-1β levels from the sera of both mice were not significantly increased. Moreover, we conducted immuno-histochemical assays on ankle joints 24 h after MSU injection. Slides prepared from WT and *caspase-11*^−/−^ mice were stained for IL-1β to examine *in vivo* production of this key gout cytokine within the joint space. Images quantified via the Aperio ImageScope™ demonstrated that *caspase-11*^−/−^ mice exhibited a significant defect in production of IL-1β protein in cells of the synovium compared to that of WT mice (Figures 5C,D). However, this difference is not due to differential cell death, since the treatment of macrophages with MSU with or without IL-1β produces minimal cytotoxicity (Supplementary Figure 4). The difference is also not due to difference in MSU phagocytosis (Supplementary Figures 5A,B). In addition, because the recognition and uptake of MSU contributes to the inflammatory response during an acute gouty attack, we determined if the absence of caspase-11 affected the phagocytosis of MSU crystals. Flow cytometric analysis from WT and *caspase-11*^−/−^ macrophages treated with MSU revealed that levels of crystal uptake were comparable between the two strains of macrophages. These data indicate that the reduction in inflammatory cytokine production was not due to an innate deficit in phagocytic capability of the *caspase-11*^−/−^ cells (Supplementary Figures 5A,B). Taken together, these data demonstrate that caspase-11 promotes the production and maturation of IL-1β during MSU injection in our gout model in a localized fashion and does not affect the phagocytosis of MSU.

### IL-1β Induces Caspase-11 Expression via IL-1R and MYD88 in Macrophages

**Figure 6:**
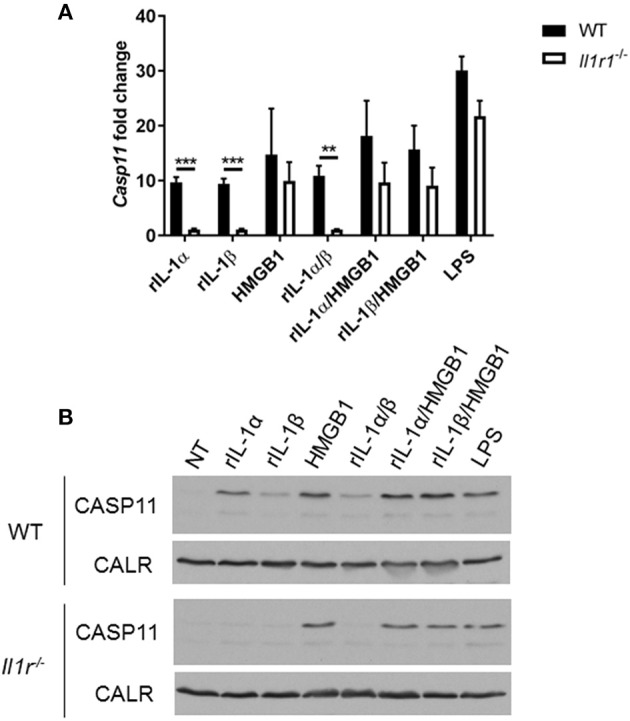
IL-1R-mediated signaling promotes caspase-11 induction *in vitro*. WT and *Il-1r1*^−/−^ macrophages stimulated with IL-1α, IL-1β, HMGB1, and LPS and in combination for 4 h. Induction of caspase-11 assayed via **(A)** RT-qPCR relative copy number and **(B)** Western with housekeeping protein calreticulin (CALR) (representative). Statistical significance was determined by using multiple *t* test with a Holm's Sidak correction. **p <0.01, ***p <0.001, *n* = 3 independent experiments.

**Figure 7:**
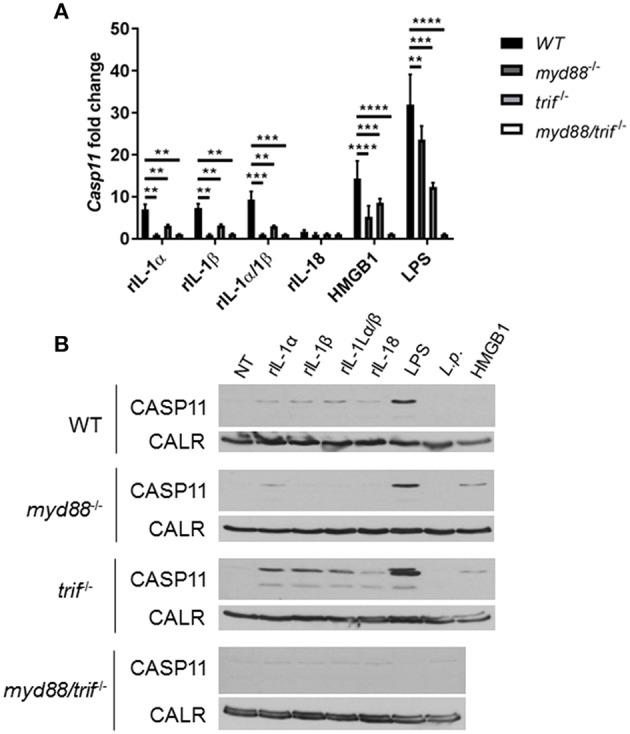
Caspase-11 induction via IL-1/IL-1R is Myd88-dependent *in vitro*. WT, *myd88*^−/−^, *trif*^−/−^, and m*yd88/trif*^−/−^ macrophages stimulated with IL-1α, IL-1β, IL-18, HMGB1, LPS, or in combination for 4 h. Induction of caspase-11 gene expression assayed via **(A)** RT-qPCR via relative copy number and **(B)** Western with loading control calreticulin (CALR) (representative). Two-way ANOVA using a Tukey's multiple comparison test was performed. **p <0.01, ***p <0.001, ****p <0.0001, *n* = 3 independent experiments.

Unlike caspase-1, caspase-11 is expressed at low levels in resting immune cells, and is induced following stimulation with various PAMPs or DAMPs (Supplementary Figures 3A,B) (43, 44). MSU treatment is accompanied by the activation of the inflammasome only in primed immune cells (11). MSU treatment alone does not induce the expression of caspase-11 (Supplementary Figures 3C,D) and is not contaminated with LPS (Supplementary Figure 6). Thus, macrophages require lipopolysaccharide (LPS) priming before MSU treatment in order to induce the expression of caspase-11 (Supplementary Figures 3C,D). Since LPS does not play a role in gout, we determined if other agents, such as cytokines, mediate the priming of immune cells in gout. To determine if the inflammatory environment of gout, specifically IL-1 cytokines, promotes caspase-11 expression, we treated macrophages with IL-1α, IL-1β, and HMGB1. Because IL-1 cytokines signal through the IL-1 receptor (R), *Il-1r1*^−/−^ macrophages were used as a control. IL-1α and IL-1β significantly upregulated caspase-11 expression in an IL-1R-dependent manner, while HMGB1 and LPS did so independently of the IL-1 receptor (Figures 6A,B). Together, these data show that the IL-1 cytokines induce caspase-11 expression via IL-1R. Surface cell receptors transmit signals from extracellular stimuli intracellularly to the nucleus via the signaling adaptors myeloid differentiation factor 88 (MYD88) and TIR domain-containing adapter-inducing interferon-β (TRIF) (45, 46). The IL-1R employs the MYD88 adaptor to transduce signals (47). To determine the requirement of MYD88 for caspase-11 expression in the presence of IL-1 cytokines, WT, *myd88*^−/−^, *trif*^−/−^, and *myd88/trif*^−/−^ macrophages were treated with IL-1α, IL-1β, IL-18, and HMGB1, and then caspase-11 expression was assessed. RT-qPCR and Western blot analysis revealed that caspase-11 mRNA and protein levels were significantly reduced in the *myd88*^−/−^ and *myd88/trif*^−/−^ cells when treated with IL-1α and IL-1β, or in combination, respectively (Figures 7A,B). HMGB1 utilizes a variety of receptors to promote its signaling events and LPS is sensed by TLR4, which uses both adaptors to signal (48–50). Subsequently, caspase-11 expression was not significantly hampered in the WT or the single knockout macrophages during stimulation with either HMGB1 or LPS, except when both MYD88 and TRIF were absent, indicating that either adapter was able to countervail for the deficient one in order to induce caspase-11 (Figures 7A,B). Together, these data indicate that IL-1α and IL-1β are able to upregulate caspase-11 via IL-1R dependent on the MYD88 adaptor. These data also suggest that IL-1α and IL-1β contribute to priming of immune cells under sterile inflammatory conditions.

### Caspase-11^−/−^ Neutrophils Migrate Less Than WT Counterparts *in vivo*

**Figure 8:**
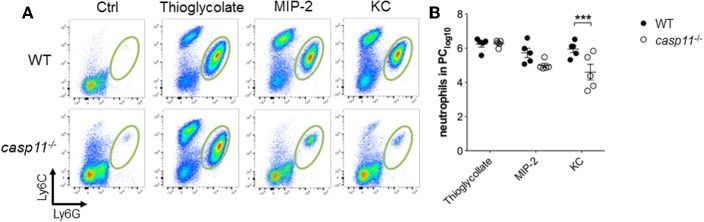
Caspase-11 promotes cellular migration *in vivo*. WT and *caspase-11*^−/−^ mice were treated with PBS, thioglycolate, CXCL1 (KC), or CXCL2 (MIP-2). At 4 h post injection, cells infiltrating into the peritoneum were isolated and analyzed via flow cytometry. **(A)** Flow plots represent the distribution of neutrophils of each group (Ly6G^high^/Ly6C^int^ neutrophils). **(B)** Graphs show total numbers of neutrophils in peritoneal cavity. A Student's *t* test was performed for statistical analysis. ***p <0.001, *n* = 5 mice.

A characteristic feature of crystal-induced gouty arthritis is the rapid accumulation of neutrophils (8, 16, 17). Chemokines that bind the IL-8 receptor CXCR-2 are essential for the development of acute neutrophilic inflammation in response to MSU in the subcutaneous air pouch model (51). Our histological data above demonstrate that significantly less inflammatory cells infiltrate the gout-afflicted joint in *caspase-11*^−/−^ mice. It is possible that the lack of inflammatory cell migration in the absence of caspase-11 is due to low cytokine and chemokine production (Figure 4 and Supplementary Figure 7). It is also possible that the lack of caspase-11 expression leads to an intrinsic defect in cell migration even in the presence of chemo-attractants. To differentiate between these two possibilities, WT and *caspase-11*^−/−^ mice were injected with KC, CXCL2 (MIP-2), and thioglycollate into the peritoneal cavity. Flow cytometry analysis of peritoneal lavages revealed that the ability of *caspase-11*^−/−^ neutrophils to migrate in response to KC was significantly reduced after 4 h of KC stimulation (Figure 8). Although the recruitment of neutrophils in response to MIP-2 was reduced in the *caspase-11*^−/−^ mice, as compared to WT mice, the difference was not statistically significant. In contrast, chemical stimulation with thioglycollate led to similar recruitment of inflammatory cells in the peritoneal cavity of *caspase-11*^−/−^ and WT mice (Figures 8A,B). It was also revealed that Ly6C^hi^ monocytes infiltrated the peritoneal cavity in significantly lower numbers in the *caspase-11*^−/−^ mouse when injected with either MIP2 or KC (Supplementary Figure 9). These results indicate that *caspase-11*^−/−^ neutrophils and monocytes are less capable to migrate in response to chemokines *in vivo*.

### Caspase-11 Regulates Directionality During Neutrophil Chemotaxis

**Figure 9:**
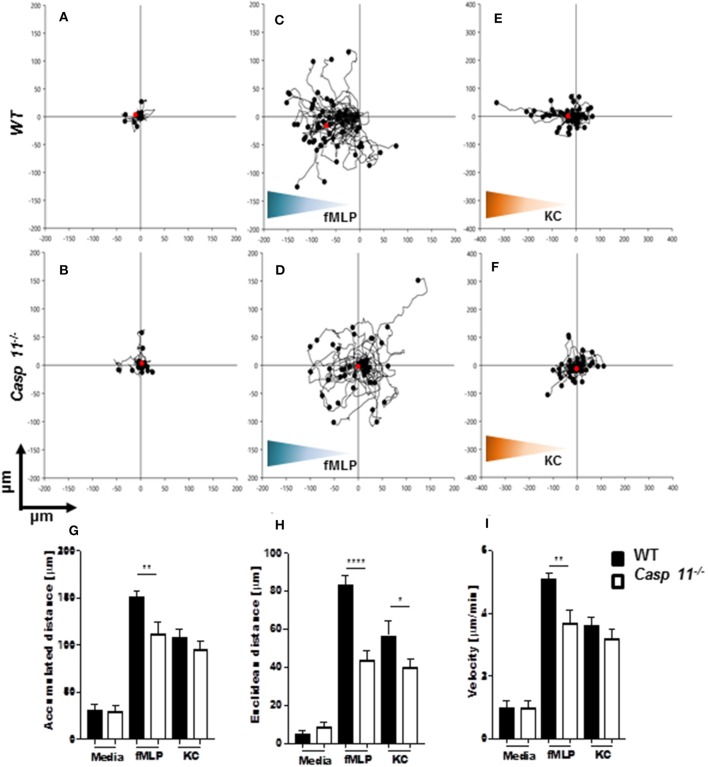
*Caspase-11*^−/−^ neutrophils display an intrinsic defect in chemotaxis *in vitro*. WT and *caspase-11*^−/−^ neutrophils were seeded in μ-slide chemotaxis chamber for 20 min, followed by the addition of the respective chemoattractant. Cell motility was recorded by video microscopy for 30 min. The start point of each track was normalized to *x* = 0 and *y* = 0, and positive *x*-axis values represent movement in the direction of the source of chemoattractant. The polar plots show the patterns of migration of WT (upper panel) or *caspase-11*^−/−^ (lower panel) neutrophils in the presence of **(A,B)** media, **(C,D)** 5 μM fMLP gradient (blue gradient), and **(E,F)** 500 ng/ml mouse recombinant KC (green gradient). The red dots indicate the average motility center of mass, (**A** and **B**, *n* = 40 cells; **C** and **F**, *n* = 60). The motility parameters of accumulated distance **(G)**, Euclidean distance **(H)**, or velocity **(I)** for WT and *caspase-11*^−/−^ neutrophils were calculated using the tracking tool PRO v2.1 software. Images were analyzed with the iBidi Chemotaxis and Migration Tool 2.0 software. The trajectories of 40 migrating cells were plotted (per treatment) and represented as rose diagrams to indicate directional integrity. The accumulated distance (μm), Euclidean distance (μm), and cellular velocity (μm/min) were calculated. Student's *t* test was performed. *p <0.05, **p <0.01, ****p <0.0001, *n* = 3 mice.

Successful chemotaxis requires not only increased motility but also sustained directionality (52, 53). In order to determine whether caspase-11 controls motility and/or directionality in response to KC, we performed time-lapse and trajectory analyses of chemotactic neutrophils *in vitro*. Bacterial peptide N-formyl-methionylleucyl- phenylalanine (fMLP) was used as a control. Neutrophils were seeded into a chemotaxis iBidi chamber that allows free cell migration in a 360° field and maintains a stable chemotactic gradient (34, 54). The migration tracks of neutrophils moving toward fMLP and KC (Figures 9A–I) were recorded, and directionality and mean velocity were analyzed (Figures 9G–I). The accumulated distance and velocity of *caspase-11*^−/−^ and WT neutrophils were similar in response to KC. However, Euclidean distance (shortest distance between start and end of migration) traveled in response to KC is significantly reduced in *caspase-11*^−/−^ neutrophils when compared to WT cells (Figure 9H). The lack of linear displacement and lack of directionality of *caspase-11*^−/−^ neutrophils, despite normal velocity in response to KC, are clear indications of a loss of efficiency in guided motility. However, all parameters including accumulated distance, Euclidean distance, and velocity are significantly reduced in *caspase-11*^−/−^ neutrophils in response to fMLP (Figures 9G–I). Together, these results demonstrate that caspase-11 is required for neutrophils to reach the site of inflammation.

### Caspase-11^−/−^ Neutrophils Produce Significantly Less NET Structures Upon Exposure to MSU When Compared to WT Cells Independently of the MLKL/RIPK3 Pathway

**Figure 10:**
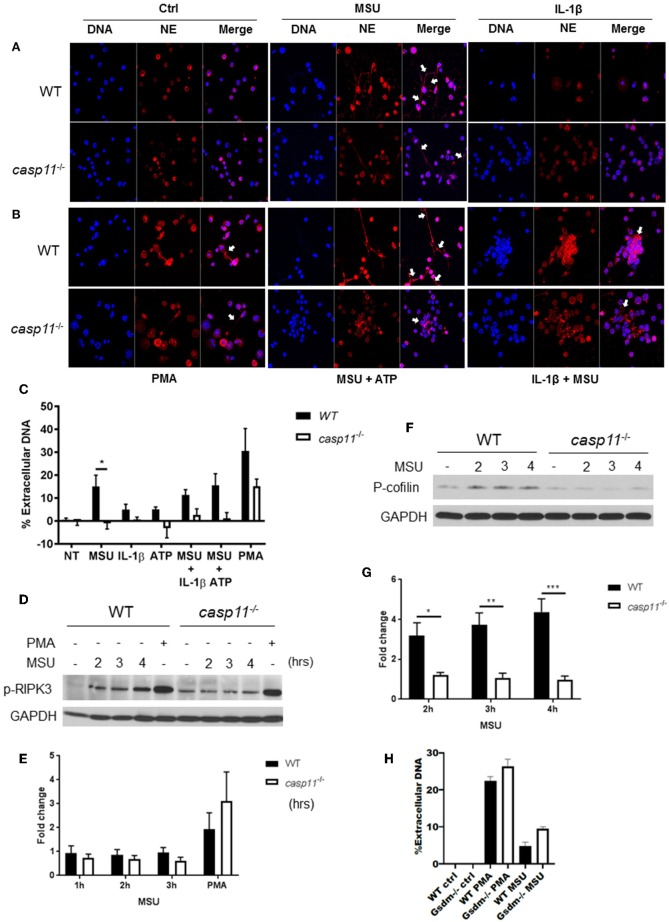
*Caspase-11*^−/−^ neutrophils produce less NETs independently of Rip3 phosphorylation and associated with altered cofilin phosphorylation in response to MSU *in vitro*. **(A,B)** Immunofluorescence assay of WT and *caspase-11*^−/−^ neutrophils treated with MSU, MSU + ATP, IL-1β, IL-1β + MSU, and PMA for 3 h. Cells were stained with neutrophil elastase (NE) and DAPI (DNA) to visualize NET formation, *n* = 3. **(C)** Quantification of NET formation *in vitro*. Response measured from WT and *caspase-11*^−/−^ neutrophils stimulated with MSU, ATP, IL-1β, combinations of treatments, and PMA. Cells were stained with Sytox green, *n* = 2 independent experiments (3 mice per group, total 6 mice), *p <0.05. **(D)** Representative Western blot for phospho-RIPK3 (p-RIPK3) during MSU treatment for 2, 3, and 4 h, *n* = 3 experiments. **(E)** Densitometry analysis of phosphorylation of Ripk3, *n* = 5. **(F)** Representative Western blot for phospho-cofilin (p-cofilin) and **(G)** densitometry analysis, *n* = 5. **(H)** Quantification of NET formation *in vitro* in response to MSU and PMA in WT and *gsdm-11*^−/−^ neutrophils. **(C,E,G,H)** Student's *t* test was performed for statistical analysis. *n* = 3 mice. *p <0.05, **p <0.01, ***p <0.001.

NETs are web-like structures formed by the mixture of chromatin with the contents of neutrophil granules including myeloperoxidase, elastase, and cathelicidins (55, 56). To determine if caspase-11 is required for NET formation by neutrophils in response to MSU, WT and *caspase-11*^−/−^ neutrophils were treated with MSU and NET structures were determined by fluorescent co-staining of extracellular DNA and neutrophil elastase (NE). *Caspase-11*^−/−^ neutrophils produced drastically less extracellular DNA and NET formation upon stimulation with MSU alone or with ATP (Figures 10A–C). Both WT and *caspase-11*^−/−^ neutrophils did not produce significant NETs when treated with ATP or IL-1β alone (Figures 10A,C). Although phorbol 12-myristate 13-acetate (PMA) is a strong inducer of NET formation, we found that NET formation by *caspase-11*^−/−^ neutrophils was reduced (Figures 10B,C). Therefore, the lack of caspase-11 reduces the production of NETs in response to different stimuli. To understand the mechanism linking caspase-11 with NET formation, we examined the phosphorylation of RIPK3 since the MLKL/RIPK3 pathway was shown to promote NET formation and necroptosis (57, 58). RIPK3 phosphorylation was not significantly reduced between WT and *caspase-11*^−/−^ neutrophils in response to MSU (Figures 10D,E). Therefore, caspase-11 but not MLKL/RIPK3 is needed for NETosis in response to MSU.

To form NETs, neutrophils release a DNA scaffold through a process that requires rearrangements in the microtubule network and F-actin. Inflammatory mediators, such as IL-8, fMLP, LPS, and TNF, can trigger NET formation (59–61). Because the actin cytoskeleton plays a major role in neutrophil migration (54), we investigated the activation status of cofilin in WT and c*aspase-11*^−/−^ neutrophils in response to MSU. Notably, WT neutrophils elicit low levels of phospho-cofilin expression when unstimulated (Figures 10F,G). Then, upon treatment with MSU, the phosphorylation increased significantly (Figure 10G). On the other hand, *caspase-11*^−/−^ neutrophils did not elicit a change in cofilin phosphorylation in response to MSU treatment when compared to WT. Of note, there was no difference in NET formation between WT and GSDM^−/−^ neutrophils in response to MSU (Figure 10H). Therefore, the lack of caspase-11 expression alters neutrophil's ability to phosphorylate cofilin, which is associated with reduced neutrophil migration to specific chemokine.

## Discussion

Innate immune cell recruitment, inflammatory cytokine release, tissue damage, and remodeling in gout have been linked to the activation of the Nlrp3 inflammasome in response to MSU (11). Plasma concentrations of IL-1β, IL-18, IL-6, and IL-8 are elevated in the gout patients (9, 13), but little is known about the role of these cytokines in the progression of gout. Additionally, many reports have focused on the role of caspase-1 in gout; however, little is known to date about the role of caspase-11 in this sterile, inflammatory disease condition. The role of caspase-11 is versatile according to the insult. It has been described to be upstream, independent, or parallel to caspase-1 activation (26, 26, 30, 62–66). When macrophages are treated with intracellular LPS, caspase-11 senses this complex molecule leading to downstream activation of caspase-1 and IL-1β (32, 67). However, when macrophages face an organism such as *L. pneumophila*, caspase-1 activation is independent of caspase-11 (25). Several reports demonstrated that caspase-11 mediates the release but not the activation of IL-1β (66). Furthermore, we demonstrated that caspase-11 modulates vesicular trafficking through altering the activation of cofilin via the Rho GTPase (24, 25). We demonstrated that after phagocytosis of *L. pneumophila*, caspase-11 modulates the phosphorylation of cofilin through upstream effector molecules. This balance in its activation promotes actin polymerization and, consequently, efficient phagolysosomal fusion and destruction of the intracellular pathogen (24, 25). Without affecting the initial uptake, the absence of caspase-11 allowed greater intracellular growth because of the defect in actin dynamics (24, 25). Because of this, we believe that caspase-11 regulates actin polymerization through cofilin, affecting subcellular organization of vesicles and organelles without affecting phagocytic events. In addition, we found that caspase-11 promotes the formation and maturation of autophagosomes (26). Recently, it was shown that caspase-11 responds to lipotechoic acid and Gram-positive bacteria, leading to activation of the inflammasome (68). In the case of methicillin-resistant *Staphylococcus aureus* (MRSA), caspase-11 is actually exploited to actively prevent the recruitment of mitochondria to the vicinity of the bacteria-containing vacuoles to avoid intracellular killing (28).

It is still not clear what specifically induces the expression of caspase-11 in immune cells during sterile inflammation. Here, we found that IL-1β and IL-1α induce caspase-11 expression via the IL-1R/MYD88 pathway. It is possible that IL-1β and IL-1α are released from dead cells with the gout-inflicted joint leading to the induction of caspase-11 in an IL-1R-dependent manner, priming immune cells for inflammasome activation by subsequent insults including MSU. Therefore, the expression of caspase-11 is induced not only by microbes but also by specific cytokines. It would be interesting to examine if other conditions where caspase-11 was induced were not due to IL-1β-mediated induction rather than directly by the PAMP or DAMP molecules.

Several prominent studies have demonstrated that caspase-11-mediated functions, including the release of IL-1β, are attributed to pyroptosis (66, 69, 70). Notably, the release of IL-1β from macrophages in response to MSU was not accompanied by cell death, as shown in our study. Our findings are corroborated by recent reports describing how Gsdm forms pores within the intact plasma membrane, allowing the release of IL-1β independently of cell death (4, 71). Additionally, we found that caspase-11 promotes the secretion of inflammasome-independent cytokines/chemokines from macrophages such as TNFα, IL-6, and KC without provoking cell death as demonstrated by the scarce amounts of LDH. Therefore, caspase-11 plays a major role in gout pathogenesis through the regulation of cytokine production independently of cell death.

Gout has been classified as an IL-1β prototypic condition since neutralizing antibodies to IL-1β or the caspase-1 inhibitor z-YVAD significantly reduce inflammation and the production of other cytokines within the joints (9, 11, 13, 18). Nevertheless, these approaches were not effective in most gout patients and could not prevent joint damage since they target extracellular IL-1β after tissue damage has occurred. Alternatively, preventing the expression of caspase-11 in gout-prone individuals may prevent the instigation of tissue damage.

Neutrophils dramatically contribute to joint injury once they migrate to the MSU deposition site (8, 16, 17). Neutrophil remnants may amplify the inflammatory response beyond their short lifespan in the tissues, contributing to chronic inflammation. Neutrophil influx is impaired in an *in vivo* model of crystal-induced peritonitis in *Nlrp3*^−/−^, *Asc*^−/−^, *Casp-1/Caspase-11*^−/−^, and *Il-1r*^−/−^ mice (11). *Asc*^−/−^ and *Casp-1/Caspase-11*^−/−^ neutrophils migrated similar to WT neutrophils in response to zymosan, but not MSU (11). The main mechanism of reduced migration has been attributed to low production of cytokines. However, the role of caspase-11 in neutrophil migration and function in gout has not been previously studied. Here, we show that neutrophil migration to MSU-injected joints is significantly reduced in the absence of caspase-11 expression. Indeed, the absence of caspase-11 is also accompanied by reduced KC production, which plays an important role in neutrophil chemotaxis. Therefore, it is possible that the reduced migration of *caspase-11*^−/−^ neutrophils is due to reduced KC production. It is also probable that the lack of caspase-11 expression in neutrophils exerts an inherent defect in the migration machinery, which was not previously tested. We demonstrate here that neutrophils lacking caspase-11 expression migrate similar to their WT counterparts in response to intraperitoneal injection of PMA or MIP2 but not KC. Nevertheless, iBidi chamber analysis showed that the accumulated distance, velocity, and directionality in response to fMLP require the expression of caspase-11. Yet, caspase-11 was essential for the directionality of neutrophils toward KC but not velocity or total distance traveled. Together these data describe a novel role for caspase-11 in neutrophil migration that is not universal for all chemoattractants, which warrant further studies.

Notably, macrophage-derived IL-1β enhances MSU crystal-induced NET formation in neutrophils (72). Neutrophils were thought to release NETs during a distinct form of cell death, named NETosis (20, 59). Current evidence, however, suggests that neutrophils may remain viable and functional even after NET extrusion and that NET formation is independent of neutrophil death (22, 73). Discovering here that caspase-11 is required for NET formation in response to MSU and to a lesser extent to PMA, IL-1β, and ATP points to the distinct and specific roles of caspase-11 in different cell types and disease conditions. It has been reported that necroptosis and NETosis require the functions of RIPK3 and MLKL protein. However, we found that this pathway is not engaged in response to MSU. Our findings support a recent report showing that NET formation occurs independently of the MLKL/RIPK3 pathway in response to PMA, LPS, and Gram-negative bacteria (74). Autophagy has also been implicated in NETs (8, 55). Of note, we have demonstrated that caspase-11 promotes autophagy in response to bacterial infection (26). Yet, reports have demonstrated that NET formation can still occur in autophagy-deficient and RIPK3 knockout neutrophils. It is clear that MSU and maybe other crystals induce NETs through a molecular pathway that is distinct from that of PMA. Interestingly, the lack of NETs in *caspase-11*^−/−^ neutrophils in response to MSU is also accompanied by lack of cofilin phosphorylation, supporting a role for caspase-11-mediated actin polymerization in NET formation. It has also been shown that NET formation plays a role in the resolution of inflammation. Aggregated NETs sequester and degrade inflammatory mediators (75). The authors point to the importance of ROS production as a main factor for NET aggregation that in turn resolve neutrophil-mediated cytokines and chemokines. In our model, *caspase-11*^−/−^ produced less NETs when treated with PMA, a strong ROS inducer. Thus, we postulate that NET formation is not solely reliant on ROS and that caspase-11-mediated regulation of the actin cytoskeleton via cofilin plays a major role. In addition, the reduction of NETs from caspase-11 PMNs would suggest an increase of inflammatory mediators. Conversely, significantly lower levels of gout cytokines were present within the articular joints of *caspase-11*^−/−^ mice, indicating that NETs could be involved in the reduction of inflammatory cytokines, but are not needed for their production. Accordingly, it is likely that caspase-11 is needed for the inflammatory cytokine production and chemotaxis in response to MSU. Additionally, it is possible that the caspase-11 downstream effector GSDM is required for NET formation in response to MSU. A recent report demonstrated that this is the case during infection with the Gram-negative bacteria *Salmonella* (76). Together, these findings demonstrate a distinct physiological NET pathway in response to MSU crystals that is independent of MLKL/RIPK3 and dependent on caspase-11.

Our findings can apply to other sterile inflammatory conditions where neutrophil migration and NET formation is significantly involved in the pathogenesis of the disease such as thrombosis, systemic lupus erythematosus (SLE) ANCA vasculitis, and other crystallopathies (73). Targeting caspase-11 is a viable possibility during or before acute gout attacks, but further studies are needed to test this option in chronic gout. Targeting caspase-11 can also be used to avert early steps of gout pathobiology before the onset of symptoms and before tissue damage occurs in prone individuals. Cumulatively, these results offer previously unrecognized functions for caspase-11 that adds to its reported diverse roles. Concurrently, we provide mechanistic evidence that caspase-11 is a viable therapeutic target in gout and other disease conditions that are inflicted by activated neutrophils.

## Materials and Methods

### Mice

Wild-type (WT) C57BL/6 mice were purchased from The Jackson Laboratory (Bar Harbor, ME). C*aspase-11*^−/−^ mice were generously donated by Dr. Yuan at Harvard Medical School (43). *Caspase-1*^−/−^ (C*aspase-1*^−/−^/*Caspase-11*^*Tg*^*)* mice were a gift from Dr. Vishva Dixit at Genentech (77). All mice were housed in a pathogen-free facility and experiments were performed with approval and in accordance with regulations and guidelines from the Institutional Animal Care and Use Committee (IACUC 2010A00000066-R3) at The Ohio State University (Columbus, OH).

### Acute Gouty Arthritis Model

MSU crystals were purchased from Invivogen and verified to be endotoxin free by LAL assay from Pierce (Supplementary Figure 6). Using Hamilton syringes, 0.5 mg of MSU crystals (tlrl-msu) was injected into the tibio-tarsal joints of mice under light anesthesia according to a previously characterized model of MSU crystal-induced ankle arthritis (78). After 24 h, mice were anesthetized and ankle joints were photographed and measured via caliper. Ankle joint aspirates were collected by flushing with 20 μl of PBS and blood was taken for serum isolation by axillary vessel incision of the right subclavian vein at the time of sacrifice. Cytokine analysis on collected serum was performed using electrochemiluminescence detection (V-PLEX Proinflammatory Panel 1 mouse kit; Meso Scale Diagnostics, Rockville, MD) per manufacturer's protocol. Tibio-tarsal joints were isolated and cut in half. One half was for histological analysis and the other half was stored in TRIzol™ reagent (Life Technologies, 15596018) for RNA/protein analysis.

### Histology and Immunohistochemistry

Hind limbs of mice were dissected at experimental endpoint and fixed in 10% neutral buffered formalin for 24 h. Tissue was then decalcified in TBD-2 (ThermoFisher, 6764003) according to the manufacturer's protocol and transferred to 70% ethanol. Following paraffin processing, blocks were cut at 4 microns, placed on positively charged slides, and fixed in cold acetone. Serial paraffin sections were used for immunohistochemistry and H&E staining as previously described (24, 79–81). Briefly, all slides were stained in Richard Allan Scientific Hematoxylin (Thermo Scientific, 7211L) and Eosin-Y (Thermo Scientific, 71211) with the Leica Autostainer ST5020 (Leica Biosystems, Buffalo Grove, IL). Immunohistochemistry/DAB staining was performed with MPO, F4/80, and IL-1β (1:200, Abcam, ab9787) with specific secondary (Abcam, ab6721). The Aperio ScanScope XT eSlide capture device (Aperio, Vista, CA) was used to digitally scan slides at 40 × magnification and Aperio Digital Image Analysis software (v9.1) was used to analyze and measure pixel intensities according to previously described methods (26, 82).

### Cell Culture

Bone marrow-derived macrophages (BMDMs) were cultured as previously described (25, 80, 81, 83–85). Caspase-11 induction was measured by treating WT macrophages with various inflammatory mediators for 4 h. 20 ng/ml IL-1α, IL-1β, IL-18, and KC (R&D Systems, 400-ML-005, 401-ML-005, 9139-IL-050, and 453-KC-050), HMGB1 (eBioscience, 34-8401-82), 2.5 μg/ml Pam3CSK4 (tlrl-pms), 5 μg/ml poly(I:C) (tlrl-picw), 100 ng/ml LPS (tlrl-eklps), 500 ng/ml flagellin (tlrl-stfla), 100 ng/ml FSL-1 (tlrl-fsl), 5 μg/ml ssRNA (tlrl-lrna40), 5 μg/ml Imiquimod (tlrl-imqs), and 100 ng/ml bacterial DNA (tlrl-ssec) (InvivoGen). Interferon induction of caspase-11 was achieved using 200 ng/ml IFNα and β (BioLegend, 752806, 581306), 50 ng/ml IFNγ (R&D Systems, 485-MI), and 100 ng/ml TNFα (R&D Systems, 410-MT-050) and LPS for 4 h, respectively. In vitro stimulation with MSU was accomplished with 100 μg/ml (Invivogen, tlrl-msu). ATP (Sigma-Aldrich) was used for 30 min at the final concentration of 5 mM. All cells were lysed and stored in TRIzol™.

### Enzyme-Linked Immuno-Sorbent Assay (ELISA)

*In vivo* samples were sent to the Analytical & Development Lab in the Ohio State University Clinical Research Center Core. Meso Scale Discovery V-PLEX mouse Pro-Inflammatory Panel 1 (K15048G) was used to analyze joint aspirate and serum samples. Fold change of cytokines from synovial fluid or that of serum was achieved by dividing cytokine levels obtained from MSU-injected ankles by those injected with PBS (24, 37).

### Lactate Dehydrogenase (LDH) Assay

*In vitro* supernatants were assessed for pyroptosis by measuring LDH release following the manufacturer's instructions (Promega CytoTox-ONE™ Homogenous Membrane Integrity Assay #G7891) (24, 25).

### Quantitative Real-Time PCR

Total RNA was isolated from cells lysed in TRIzol™ via phenol-chloroform extraction. *Caspase-11* and *Il-1b* mRNA expression was assessed using iQ SYBR Green Supermix (Bio Rad, 64105458). Briefly, C_t_ values of each target gene were subtracted from the average C_t_ of the housekeeping genes, *Gapdh* and *Cap-1*, and the resulting ΔC_t_ was used to calculate gene expression in relative copy numbers (RCN) as we described earlier (37, 83, 86, 87).

### Immunoblotting

Protein extraction from macrophages was performed using TRIzol™ reagent according to the manufacturer's instructions. Briefly, after phase separation using chloroform, 100% ethanol was added to the interphase/phenol-chloroform layer to precipitate genomic DNA. Subsequently, the phenol-ethanol supernatant was mixed with isopropanol in order to precipitate out proteins. Isolated protein was then denatured in a urea-based lysis buffer. The Bradford method was used to determine protein concentrations. Equal amounts of protein were separated by 12% SDS-PAGE (Biorad, 161-0158) and transferred to a polyvinylidene fluoride (PVDF) membrane (Biorad, 162-0177). Membranes were incubated overnight with antibodies against caspase-11 (Sigma, C1354-.2ML), calreticulin (Enzo Life Sciences, ADI-SPA-601-D), phospho-cofilin (Cell Signaling, #3313S), and RIP3. Corresponding secondary antibodies conjugated with horseradish peroxidase and in combination with enhanced chemiluminescence reagent were used to visualize protein bands. Densitometry analyses were performed by normalizing target protein bands to the loading control (calreticulin) using ImageJ software as previously described (24, 25, 87).

### NET Formation Assay

Bone marrow was isolated from the femur and tibia of 6- to 8-week-old WT and *caspase-11*^−/−^ mice (88). Neutrophils were negatively selected by using the EasySep™ mouse neutrophil enrichment kit (STEM cell technologies, #19762A), and 10^5^ neutrophils were plated in each well of a 96-well black plate (Corning, #3603). PMNs were stimulated for 3 h with 100 μg/ml MSU (Invivogen, tlrl-msu), 100 μM ATP (Roche, 10 519 987 001), 25 ng/ml IL-1β (R&D systems, 401-ML-005/CF), and 100 nM PMA (Sigma-Aldrich, #P8139-10MG). To remove background fluorescence, designated wells were treated with DNAse I (Sigma, #AMPD1-KT). One percent of Triton X-100 (Fisher Scientific, #BP151-100) was added to some wells to adjust the DNA staining to 100%. Before end of assay, wells were treated with 4 μM Sytox green (Life Technologies, #S7020) to stain extracellular DNA. Fluorescence was assayed on a 96-well plate reader (Spectra Max i3X, Molecular Devices). The quantification of extracellular DNA in neutrophils has previously been described (19, 88).

### Immunofluorescence

PMNs were negatively selected by using the EasySep™ mouse neutrophil enrichment kit (STEM cell technologies, #19762A); 2 ×10^5^ cells were plated in a 24-well plate on poly-L-lysine coverslips and allowed to equilibrate/adhere for 10 min. PMNs were then stimulated for 3 h with 200 nM PMA (Sigma-Aldrich, #P8139-10MG), 100 μg/ml MSU (Invivogen, tlrl-msu) alone or in combination with 100 μM ATP (Roche, 10 519 987 001), or 25 ng/ml IL-1β (R&D Systems, 401-ML-005/CF) to induce NET formation. Cells were then fixed with 10% neutral buffered formalin (NBF, Sigma, #HT501128-4L) overnight. The next day, the coverslips were blocked and permeabilized with 5% goat serum and 0.1% Triton X-100 for 1 h. Anti-neutrophil elastase antibody (Abcam, #ab21595) was used to visualize NETs (1:200 dilution), and DNA was stained with 1 μg/ml 4′-6′diamino-2-phenylindole (DAPI, Molecular Probes, #D1306). Goat anti-rabbit Alexa 594 (ThermoFisher, #R37117) was used as the secondary. Images were captured using a laser scanning confocal fluorescence microscope (Olympus Fluoview FV10i) using a 60 × objective as we previously describe (24–26).

### Peritoneal Cavity *in vivo* PMN Migration Model

The peritoneal cavity model of PMN migration has previously been described (89). Briefly, CXCL1/KC (R&D, 453-KC-050), CXCL2/MIP-2 (R&D, 452-M2-050), and 4% of thioglycolate (Fluka Analytical, 70157) were injected intraperitoneally into 8- to 10-week-old WT and *caspase-11*^−/−^ mice. Four hours after treatment, mice were euthanized and their peritoneal cavities were lavaged with 10 ml PBS + 1 mM EGTA (PBS—Life Technologies, #14190-144; EGTA—Boston Bioproducts, #BM-151). Next, red blood cells were lysed (MACS, #130-094-183), and cells were washed and stained for various cellular, surface markers including anti-CD45 (BioLegend, #103138), anti-CD11b (BioLegend, #101243), anti-Ly6G (BioLegend, #127639), anti-Ly6C (BioLegend, #128031), anti-F4/80 (BioLegend, #123147), and LIVE/Dead™ Fixable Blue dead cell stain (ThermoFisher, #L34962). Relative cell counts were assessed via flow cytometry using countBright absolute counting beads (ThermoFisher, #C36950). Gating strategy for neutrophils: single cells/live cells/CD45+/CD11b^high^/Ly6G^high^ Ly6C^int^.

### Chemotaxis Assay

Neutrophils were isolated from WT and *caspase-11*^−/−^ mice and resuspended in HBSS plus 2% FBS media. Cells were seeded into the narrow channel of μ-slide chemotaxis chamber 2D (Ibidi) for 20 min at 37°C, and then non-adherent cells were washed twice with media. The narrow channel (observation area) connects two 40-μl reservoirs. To form the chemotaxis gradient, the reservoir at the right of the chamber was filled with media and the left reservoir was filled with 5 μM fMLP (Sigma-Aldrich) or 500 ng/ml KC (R&D). The cell migration was video recorded using bright-field microscopy (Nikon Eclipse Ti). Images were captured every 30 s for 30 min, maintaining the chamber at 37°C. The chemotaxis and migration tracks analyses were performed using the Tracking Tool Pro v 2.1 software (Gradientech). Forty to 60 randomly selected cells were manually tracked per condition in each chemotaxis experiment.

### Phagocytosis of MSU Crystals

BMDMs from WT and *caspase-11*^−/−^ mice were seeded 1 ×10^6^ cells in a 6-well plate. Macrophages were allowed to rest overnight and then treated for 16 h with 100 μg/ml MSU to allow for uptake of crystals. Cells were isolated and then stained with F4/80 (Invitrogen # 12-4801-82, clone BM8) to confirm macrophage marker expression and also with LIVE/DEAD Fixable Near-IR Dead Cell stain (Invitrogen #L10119) to differentiate live and dead cells. For phagocytosis, cells were assayed on BD FACS Canto II for increase in side scatter (i.e., internal complexity/granularity) and analyzed using FlowJo™ v10.

### Measuring Endotoxin in MSU

MSU was purchased from InvivoGen and tested for the absence of endotoxin contamination by LAL Chromogenic Endotoxin Quantitation kit (Pierce) according to the manufacturer's suggestions. Briefly, standard curve was created using *E. coli* endotoxin at 1, 0.5, 0.25, and 0.1 EU/ml. Endotoxin-free water was used as a negative control and MSU was used as an unknown sample. Samples were measured on a microplate absorbance reader at 405 nm.

### Statistical Analysis

Data in figures are presented as mean± SE (standard error). At least three independent experiments were used as listed in the figure legends. Two sample *t* tests (for two group comparisons such as experiments in Figures 1B, 3B,D, 5D, 8B, 9, 10) or ANOVA (for multiple group comparisons such as experiments in Figures 1A, 4, 5A,B, 7) were used for most of the experiments as detailed in the figure legends. Tukey's or Holm's Sidak methods were used to adjust for multiple comparisons to control Type I error at α = 0.05. *p*-values ≤ 0.05 after adjustment for multiple comparisons were considered as significant. Data were analyzed using GraphPad Prism 7 software.

## Data Availability Statement

All datasets generated for this study are included in the article/Supplementary Material.

## Ethics Statement

The animal study was reviewed and approved by OSU IACUC.

## Author's Note

The content is solely the responsibility of the authors and does not necessarily represent the official views of the National Center for Advancing Translational Sciences or the National Institutes of Health.

## Author Contributions

KC designed, performed, analyzed, and interpreted data and wrote, edited, and reviewed the manuscript. NY assisted in performing *in vivo* experiments and contributed to writing and reviewing the manuscript. FR-A, KK, AA, KH, AB, AV, KD, HG, MA, ME, DD, SA, SE, SP-S, and MG contributed by performing experiments and editing the manuscript. WJ helped conceptualize experiments and interpreted data. XZ performed statistical analysis. AOA obtained funding and resources, assisted in the experimental design and implementation, interpretation of data, as well as the writing and editing of the manuscript.

### Conflict of Interest

The authors declare that the research was conducted in the absence of any commercial or financial relationships that could be construed as a potential conflict of interest.
