# Supplementary material for: Caspase-11 Mediates Neutrophil Chemotaxis and Extracellular Trap Formation During Acute Gouty Arthritis Through Alteration of Cofilin Phosphorylation
Source: Front Immunol. 2019 Nov 15;10:2519. doi: 10.3389/fimmu.2019.02519 (PMC6874099; doi:10.3389/fimmu.2019.02519)
Supplement: Supplementary file 1 [file Data_Sheet_1.pdf]

Supp Fig. 1

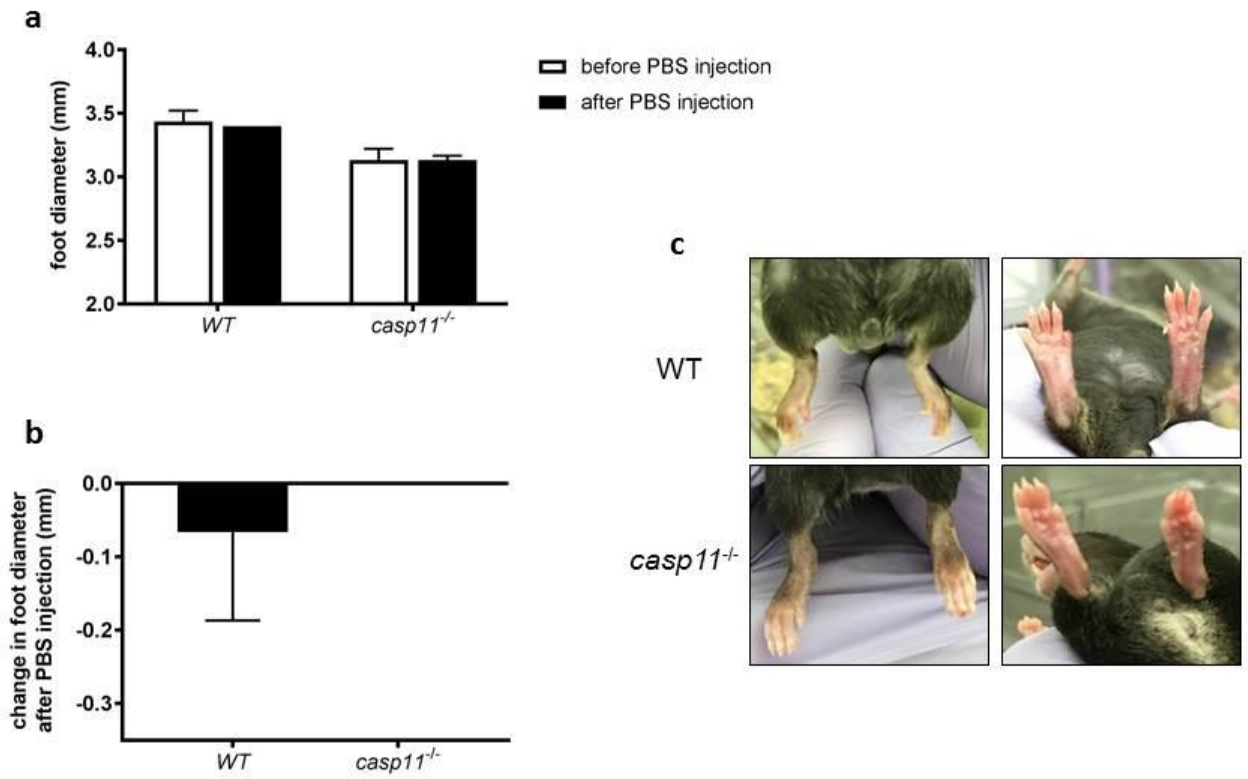
a
4.0
3.5
3.0
2.5
2.0
foot diameter (mm)
WT
casp11-/-
before PBS injection
after PBS injection
b
0.0
-0.1
-0.2
-0.3
change in foot diameter after PBS injection (mm)
WT
casp11-/-
c
WT
casp11-/-

Supp Fig. 2

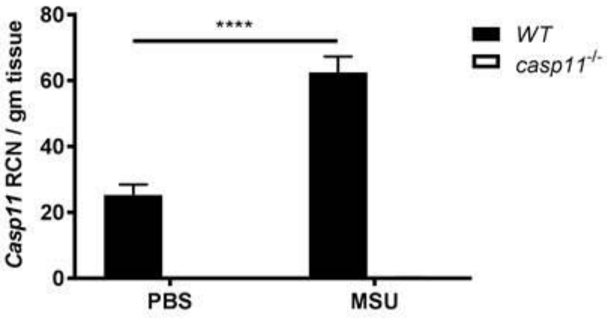

Supp Fig. 3

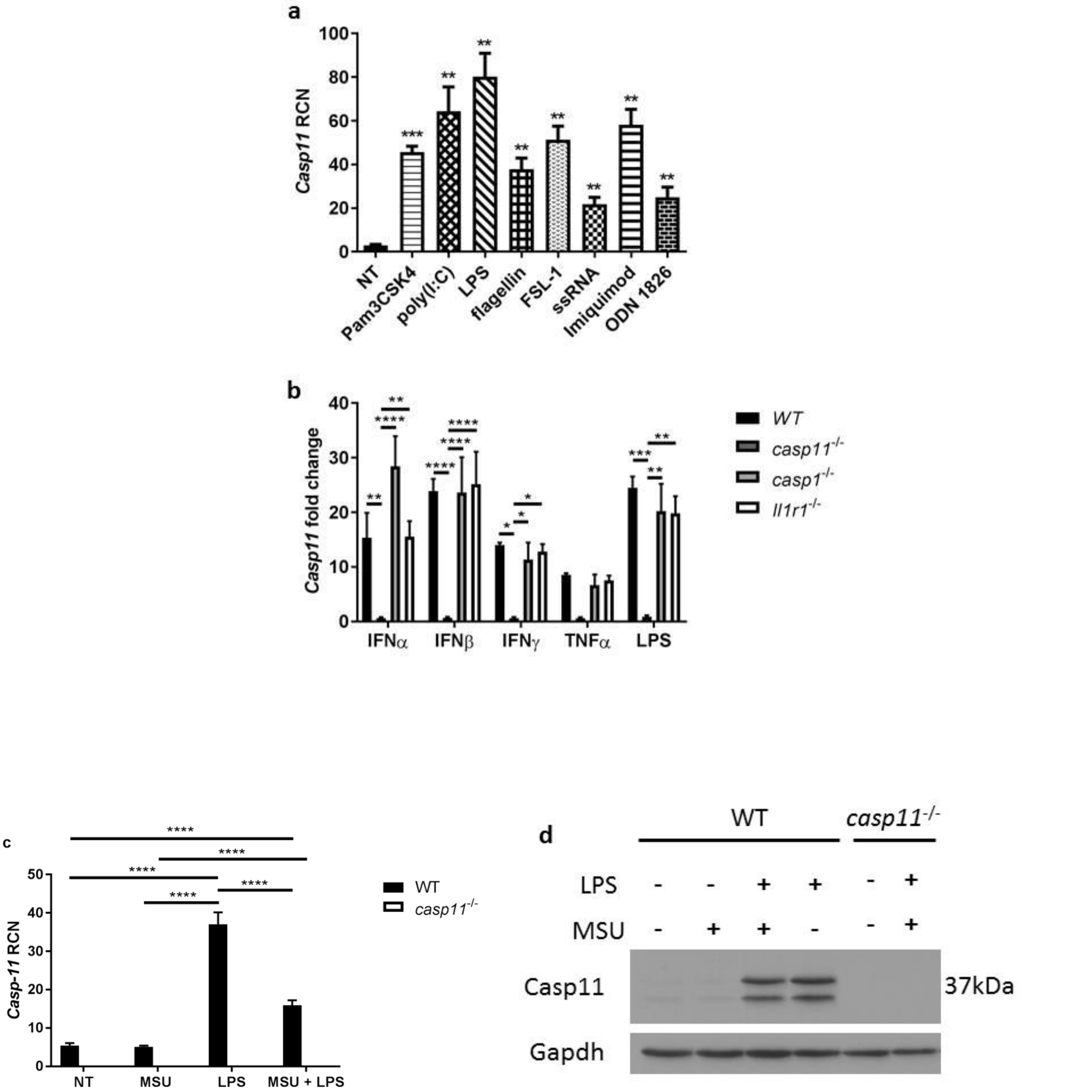
a
Casp11 RCN
100
80
60
40
20
0
***
**
NT
Pam3CSK4
poly(I:C)
LPS
flagellin
FSL-1
ssRNA
Imiquimod
ODN 1826
b
Casp11 fold change
40
30
20
10
0
**
****
***
*
IFNα
IFNβ
IFNγ
TNFα
LPS
WT
casp11-/-
casp1-/-
Il1r1-/-
c
Casp-11 RCN
50
40
30
20
10
0
****
NT
MSU
LPS
MSU + LPS
WT
casp11-/-
d
WT
casp11-/-
LPS
MSU
Casp11
37kDa
Gapdh

Supp Fig. 4

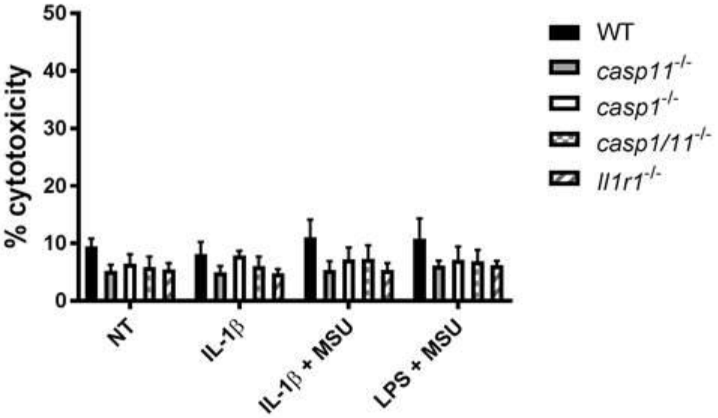

% cytotoxicity
50
40
30
20
10
0
NT
IL-1β
IL-1β + MSU
LPS + MSU
WT
casp11-/-
casp1-/-
casp1/11-/-
Il1r1-/-

Supp Fig. 5

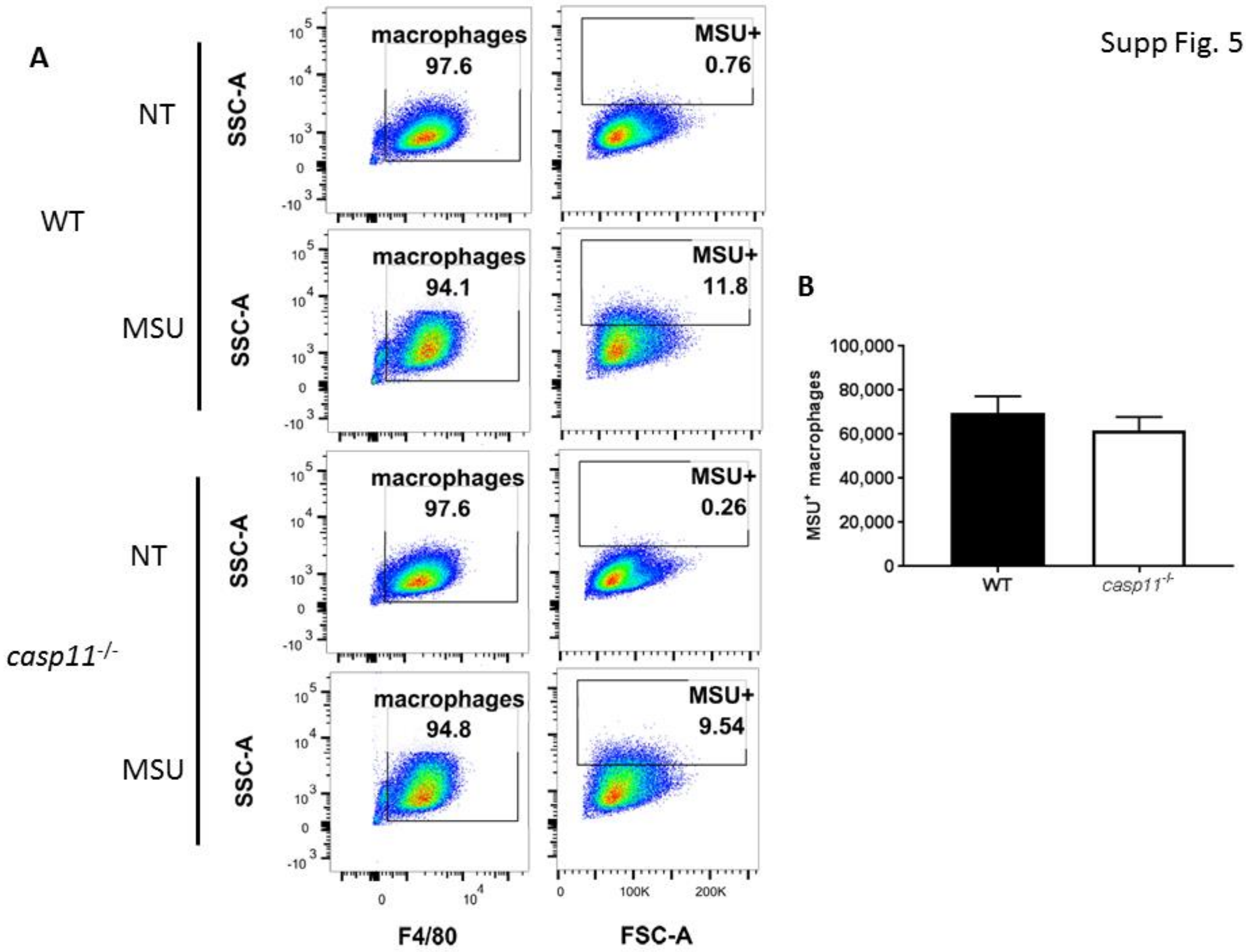

A
WT
NT
MSU
casp11-/-
NT
MSU
SSC-A
macrophages
97.6
94.1
97.6
94.8
MSU+
0.76
11.8
0.26
9.54
F4/80
FSC-A
0
10^4
100K
200K
B
MSU+ macrophages
100,000
80,000
60,000
40,000
20,000
0
WT
casp11-/-

Supp. Fig. 6

EU/ml	Absorbance @ 405 (1.0s)	Absorbance @ 630 (0.1s)	Dual Matrix Expression	Blank corrected	Average
1	1.735	0.048	1.687	1.628	
1	1.801	0.046	1.755	1.696	1.662
0.5	1.103	0.044	1.059	1.000	
0.5	1.168	0.042	1.126	1.067	1.033
0.25	0.495	0.042	0.453	0.394	
0.25	0.527	0.044	0.483	0.424	0.409
0.1	0.180	0.041	0.139	0.080	
0.1	0.195	0.042	0.152	0.093	0.087
water	0.115	0.055	0.059	0.000	
water	0.131	0.071	0.060	0.001	
MSU	0.159	0.104	0.055	-0.004	
MSU	0.089	0.042	0.046	-0.013	

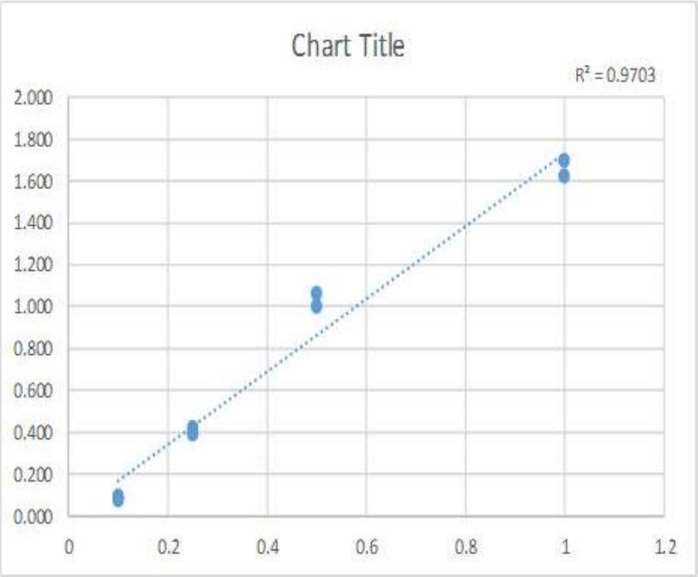

Supp Fig. 7

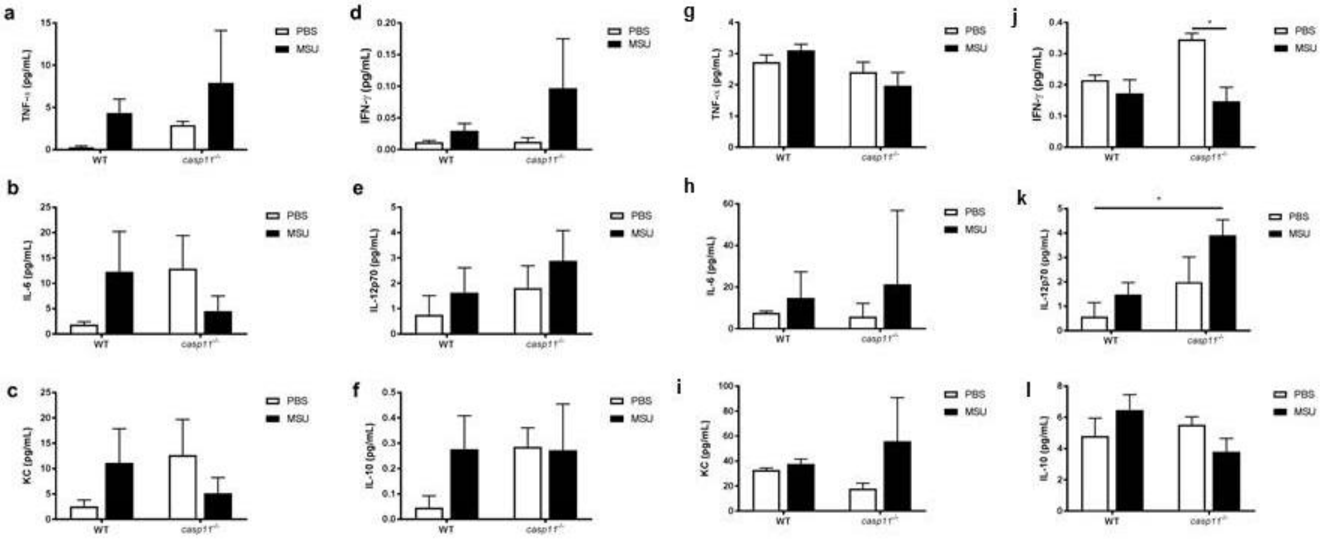
a
b
c
d
e
f
g
h
i
j
k
l
PBS
MSU
WT
casp11-/-
TNF-α (pg/mL)
IFN-γ (pg/mL)
IL-6 (pg/mL)
IL-12p70 (pg/mL)
KC (pg/mL)
IL-10 (pg/mL)

Supp Fig. 8

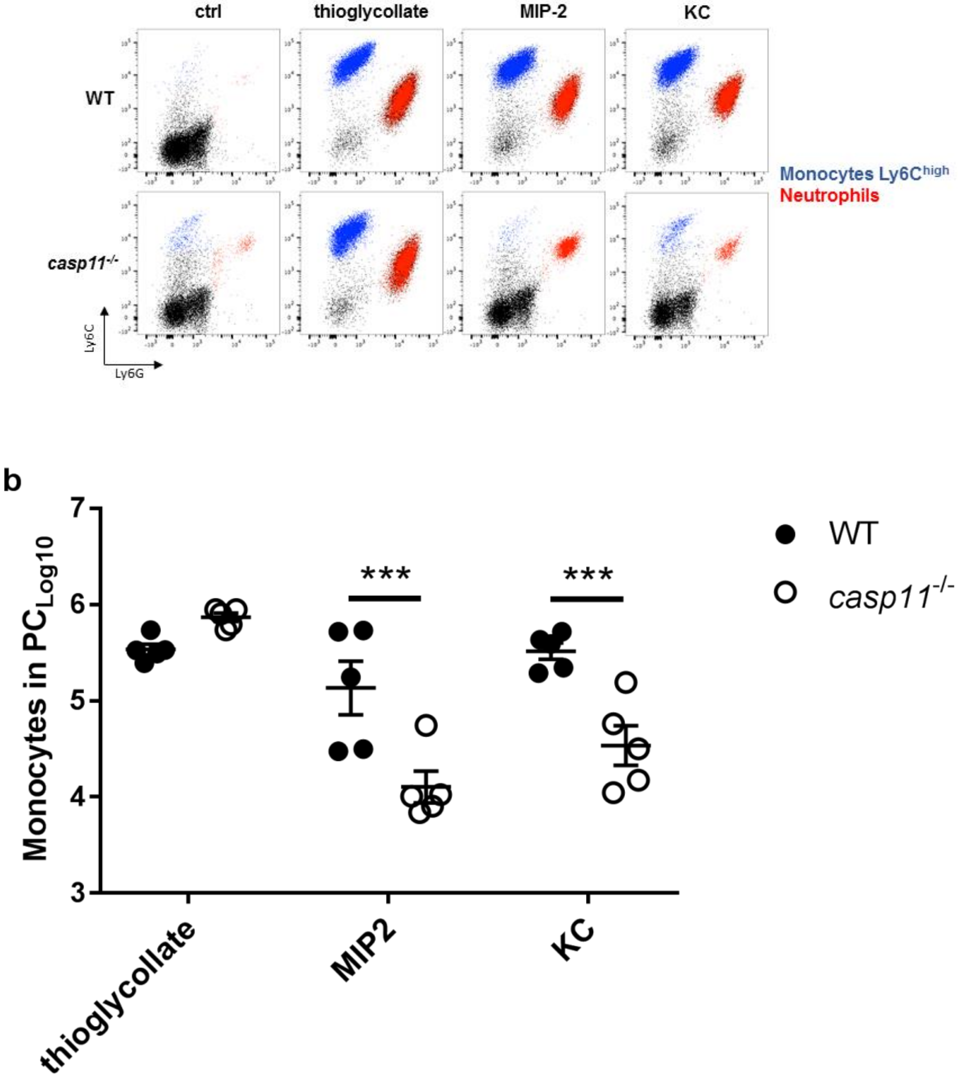
ctrl
thioglycollate
MIP-2
KC
WT
casp11-/-
Monocytes Ly6Chigh
Neutrophils
Ly6C
Ly6G
b
Monocytes in PCLog10
7
6
5
4
3
***
***
thioglycollate
MIP2
KC
WT
casp11-/-
