# Supplementary material for: Caspase-11 Mediates Neutrophil Chemotaxis and Extracellular Trap Formation During Acute Gouty Arthritis Through Alteration of Cofilin Phosphorylation
Source: Front Immunol. 2019 Nov 15;10:2519. doi: 10.3389/fimmu.2019.02519 (PMC6874099; doi:10.3389/fimmu.2019.02519)
Supplement: Supplementary file 2 [file Data_Sheet_2.docx]

**Caspase-11 mediates neutrophil chemotaxis and extracellular trap formation**

**during acute gouty arthritis through alteration of cofilin phosphorylation**

Kyle Caution^1,8^, Nicholas Young^2,8^, Frank Robledo-Avila^3^, Kathrin Krause^1,8^, Arwa Abu-Khweek^1,4,8^, Kaitlin Hamilton^1,8^, Asmaa Badr^1,8^, Anup Vaidya^1,8^, Kylene Daily^1,8^, Hawin Gosu^1,8^, Midhun Anne^1,8^, Mostafa Eltobgy^1,8^, Duaa Dakhlallah^5^, Sudha Argwal^2,8^, Shady Estfanous^1,8^, Xiaoli Zhang^7,8^, Santiago Partida-Sanchez^3^, Mikhail A. Gavrilin^7,8^, Wael N. Jarjour^2,8^, and Amal O. Amer^1,8^

**Legends for Supplemental Figures**

**Supp. Figure 1:** **Vehicle injection does not cause swelling or inflammation *in vivo*. (a)** Caliper measurement of WT and *caspase-11*^-/-^ ankle diameters before and after injection of 20uL PBS into tibio-tarsal joint. (**b)** Caliper measurement before PBS injection subtracted from measurement after injection to depict change in ankle diameter. Student’s *t*-test determined no significant difference between the strains of mice. (**c)** Representative superior and posterior pictures of WT and *caspase-11*^-/-^ ankles 24h post vehicle injection. n = 3 mice.

**Supp. Figure 2: Caspase-11 is induced *in vivo* during acute gout**. RT-qPCR analysis from WT and *caspase-11*^-/-^ joint tissue. RCN per gram of tissue. Statistical analysis performed using a Two Way ANOVA with a Tukey’s post hoc test. **** p < 0.0001, n = 5 mice.

**Supp. Figure 3: PAMPs induce Caspase-11 *in vitro*.** Expression of caspase-11 and caspase-1 RCN assessed via RT-qPCR. (**a**) WT macrophages stimulated with specific Toll-like receptor PAMPs for 4 h. One way ANOVA using Bonferroni’s correction (**b**) WT, *caspase-11*^-/-^, *caspase-1*^-/-^, and *Il-1r1*^-/-^ macrophages stimulated with IFNα, β, γ, TNFα, and LPS for 4h. Two way ANOVA with Dunnett’s comparison test. (**c**) WT and *caspase-11*^-/-^ BMDMs stimulated with 100µg/mL MSU crystals for 16hrs. Cells were Trizol lysed and isolated RNA was analyzed for relative caspase-11 expression. Two way ANOVA with Tukey’s multi comparison test. * p < 0.05, ** p < 0.01, *** p < 0.001, **** p < 0.0001. n = 3 independent experiments. (**d**) Western blot analysis of caspase-11 expression in WT and *caspase-11*^-/-^ macrophages stimulated with MSU, LPS, or both for 16hrs. Equal amounts of protein was examined using monoclonal antibodies to Gapdh. Blots are representative of three mice.

**Supp. Figure 4: MSU with or without IL-1β cause minimal cell death.** WT, *caspase-11*^-/-^, c*aspase-1*^-/-^, and *caspase-1/11*^-/-^ macrophages were treated with IL-1β with and without MSU, LPS with MSU, and LDH release was measured in their supernatants. n = 3 independent experiments. Analysis was a Two Way ANOVA with Tukey’s multi-comparison test.

**Supp. Figure 5: Quantification of monosodium urate crystal (MSU) phagocytosis**. WT and *casp11*^-/-^ bone marrow-derived macrophages were cultured and treated overnight (16hrs) with 100µg/mL MSU. Cells were collected, stained for the F4/80 surface marker, and analyzed on a BD FACS CANTO II. (**a**) Representative forward (FSC) and side (SSC) scatter flow plots of WT and *casp11*^-/-^ macrophages resting or those treated with MSU. Macrophages positive for the F4/80 marker were gated upon and then analyzed for MSU uptake indicated by an increase in the side scatter profile. Cells that moved into the upper gate, relative to the non-treated cells, indicate macrophages that phagocytosed MSU crystals. (**b**) Quantification of MSU-positive WT and *casp11*^-/-^ macrophages. The number of MSU+ cells was achieved by multiplying the frequencies of MSU+ cells relative to the live cell gate to the starting number of cells analyzed. Student’s *t*-test, n = 3 biological replicates.

**Supp. Figure 6: MSU crystals are negative for contaminating endotoxin.** Using the LAL Chromogenic Endotoxin Quantitation kit (Pierce) according to the manufacturer’s recommendations, MSU endotoxin levels were determined. Using LPS derived from *E. coli* to generate the standard curve, MSU demonstrated below detectable amounts of LPS.

**Supp. Figure 7:** *In vivo* MULTI-ARRAY electrochemiluminescence ELISA of WT and *caspase-11*^-/-^ joint aspirate (**a-f**) and serum (**g-l**) cytokine levels. (**a & g**) TNFα (**b & h**) IL-6 (**c & i**) KC (**d & j**) IFNγ (**e & k**) IL-12p70 (**f & l**) IL-10. Two Way ANOVA with a Tukey’s multi-comparison post hoc test was used to analyze statistical significance. * p < 0.05, ** p < 0.01, *** p < 0.001, **** p < 0.0001, n = 5 mice.

**Supp. Figure 8:** Caspase-11 promotes cellular migration *in vivo.* WT and *caspase-11*^-/-^ mice were treated with PBS, thioglycolate, CXCL2 (MIP-2), or CXCL1 (KC). At 4h post injection, cells infiltrating into the peritoneum were isolated and analyzed via flow cytometry. (**a**) Flow plots represent the distribution of monocytes from each group (Ly6C^high^/Ly6G^low^ monocytes). (**b**) Graphs show total numbers of neutrophils in peritoneal cavity. A Two Way ANOVA with a Holm’s Sidak post hoc test was performed for statistical analysis, *** p < 0.001, n = 5 mice.
